# Non-Thermal Plasma Treatment Diminishes Fungal Viability and Up-Regulates Resistance Genes in a Plant Host

**DOI:** 10.1371/journal.pone.0099300

**Published:** 2014-06-09

**Authors:** Kamonporn Panngom, Sang Hark Lee, Dae Hoon Park, Geon Bo Sim, Yong Hee Kim, Han Sup Uhm, Gyungsoon Park, Eun Ha Choi

**Affiliations:** 1 Plasma Bioscience Research Center, Kwangwoon University, Seoul, Republic of Korea; 2 Plasma Display Panel Research Center, Kwangwoon University, Seoul, Republic of Korea; 3 Department of Plasma Bioscience and Display, Kwangwoon University, Seoul, Republic of Korea; 4 Department of Electronic and Biological Physics, Kwangwoon University, Seoul, Republic of Korea; Yonsei University, Korea, Republic Of Seoul

## Abstract

Reactive oxygen and nitrogen species can have either harmful or beneficial effects on biological systems depending on the dose administered and the species of organism exposed, suggesting that application of reactive species can possibly produce contradictory effects in disease control, pathogen inactivation and activation of host resistance. A novel technology known as atmospheric-pressure non-thermal plasma represents a means of generating various reactive species that adversely affect pathogens (inactivation) while simultaneously up-regulating host defense genes. The anti-microbial efficacy of this technology was tested on the plant fungal pathogen *Fusarium oxysporum f.sp. lycopersici* and its susceptible host plant species *Solanum lycopercicum*. Germination of fungal spores suspended in saline was decreased over time after exposed to argon (Ar) plasma for 10 min. Although the majority of treated spores exhibited necrotic death, apoptosis was also observed along with the up-regulation of apoptosis related genes. Increases in the levels of peroxynitrite and nitrite in saline following plasma treatment may have been responsible for the observed spore death. In addition, increased transcription of pathogenesis related (PR) genes was observed in the roots of the susceptible tomato cultivar (*S. lycopercicum*) after exposure to the same Ar plasma dose used in fungal inactivation. These data suggest that atmospheric-pressure non-thermal plasma can be efficiently used to control plant fungal diseases by inactivating fungal pathogens and up-regulating mechanisms of host resistance.

## Introduction

Reactive oxygen and nitrogen species (ROS and RNS) have been shown to pose a broad spectrum of biological functions (from harmful to beneficial) depending on the dose and the species of organism exposed [1]–[4]. Phagocytic immune cells produce significant amounts of ROS and RNS in response to infectious agents as a means of inactivating pathogens [5]. At high doses, reactive species have been shown to induce apoptosis (programmed cell death) or necrosis in various mammalian cell types [6]. Conversely, ROS can also induce differentiation, migration, and proliferation of mammalian cells [7]–[9]. In eukaryotic microorganisms such as fungi, ROS regulates hyphae growth and differentiation of conidia and fruiting bodies [10]–[13], and fungal development is associated with changes in ROS levels [14]. In addition, nitric oxide (NO) functions as an important signaling molecule in living cells regulating muscle relaxation, apoptosis, disease resistance, sporulation, secondary metabolism, and sexual development [15]–[18].

The functional diversity of different reactive species can be applied to solving various biological problems. Administration of atmospheric-pressure non-thermal plasma has been demonstrated to effectively inactivate both microorganisms and cancer cells in addition to promoting wound healing and tissue regeneration [19]. Plasma is generated by subjecting gas to high energy (electric voltage) at atmospheric pressure that ionizes the gas resulting in the formation of reactive species, electrons, and ultraviolet light (>300 nm) [20]. Since various reactive species are produced from plasma, beneficial or detrimental effects on different cell types has been observed including proliferation, selective killing of cancer cell lines, or inactivation of microorganisms [21]–[26]. In addition, plasma has been used to effectively decontaminate seeds and enhance seed germination and plant growth [27]–[29]. However, application of plasma technology to treat plant infections has been rarely explored. Efficient control of fungal pathogens infecting crops requires fungal inactivation that does not harm the plant or can increase the plant's resistance to infection.

Vascular wilt disease caused by the soil-borne fungal pathogen *Fusarium oxysporum f.sp. lycopersici* affects tomato (Solanum lycopersicum) plant production resulting in significant economic losses [30]. Classical methods for controlling vascular wilt disease include the use of resistant plant cultivars, administration of antifungal agents, and bacterial biocontrol systems [30]–[33]. However, *F. oxysporum* is highly resistant to antifungal agents [32] and its plant host range has significantly expanded [30] making this approach less effective. Because the demand for more effective and environmentally friendly technologies (that are not a risk for selecting anti-fungal resistant strains) is increasing, novel therapies such as plasma may be alternative approaches that meet these requirements.

In this study, we analyzed the potential of atmospheric-pressure non-thermal dielectric barrier discharge (DBD) plasma to inactivate *F. oxysporum f.sp. lycopersici* spores in addition to assessing its effects on the host *S. lycopersicum* plant. Data presented in this report demonstrated that plasma produced two different effects: inactivation of *F. oxysporum* spores and activation of disease resistance genes in treated tomato plants.

## Materials and Methods

### Fungus and host plant culture conditions

*F. oxysporum f.sp. lycopersici* race 2 (KACC 40037), the causative fungal agent resulting in vascular wilt disease, was used in this study. The fungus was cultured on potato dextrose agar (PDA) medium (MB cell, Los Angeles, CA, USA) at 28°C in the dark. Sporulation was induced in 100 ml of Vogel Minimal (VM, [34]) liquid inoculated with pieces of fungal mycelia grown on PDA and incubated at 28°C with shaking for 4 days. Fungal spores were then collected by filtering liquid cultures through 4 layers of sterile Miracloth (Calbiochem, Darmstardt, Germany). Filtered liquid was centrifuged at 4000 rpm for 5 min and resuspended in either PBS or saline.

*Solanum lycopersicum* (a tomato cultivar named “titichal” [35], Nongwoo Bio, Suwon, Korea) was used as the plant host for *F. oxysporum*. Tomato seeds planted in vermiculite containers were maintained in a growth chamber (Hanbaek Scientific Co., Gyeonggi-do, Korea) at 25°C and exposed to 16 h light and 50% humidity. After germination, plants were grown for 2 weeks before treated with plasma.

### Plasma device and treatment

**Figure 1:**
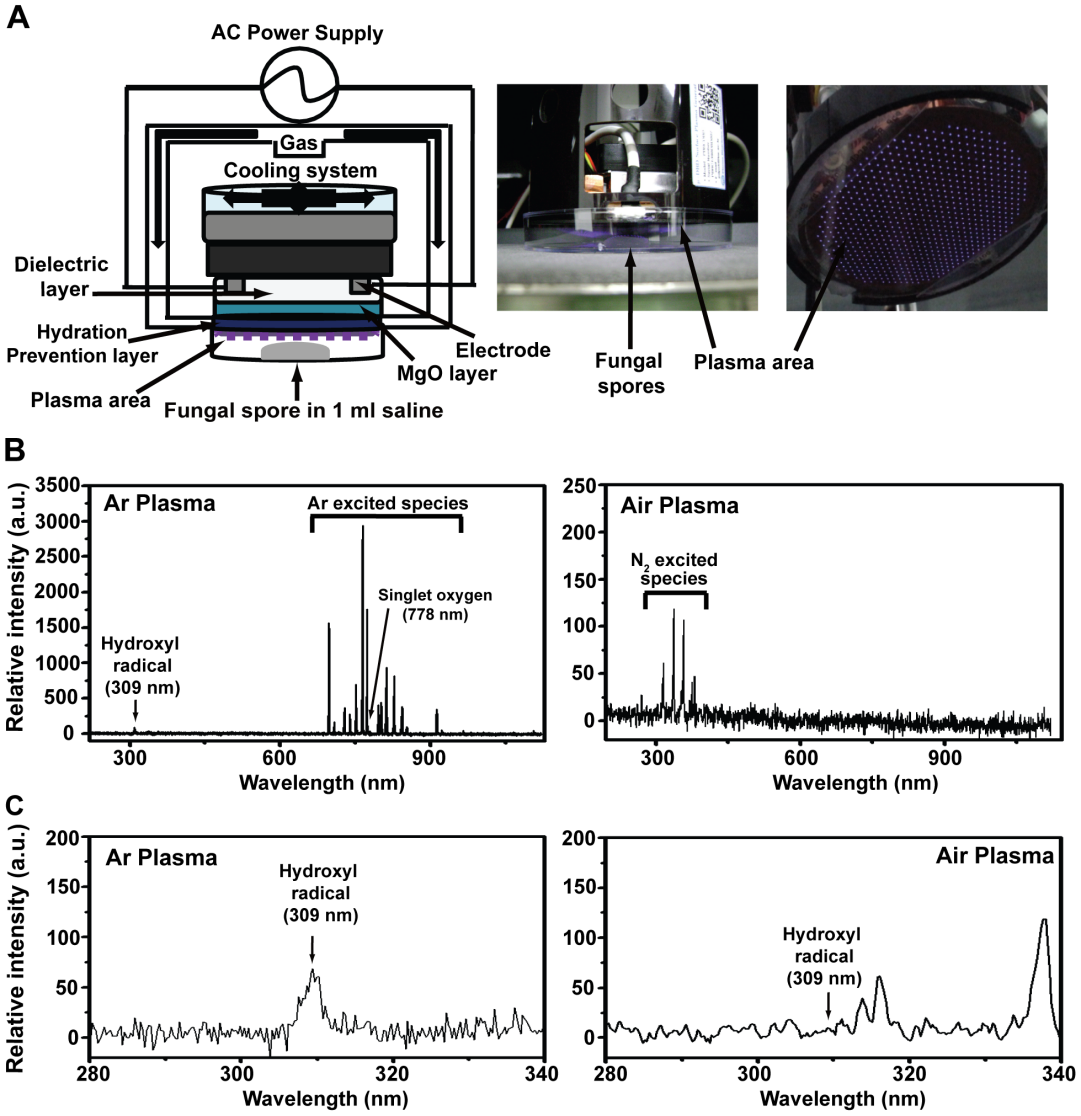
The non-thermal DBD plasma device and optical emission spectroscopy. **A**, Schematic diagram of the plasma device (left panel), experimental set-up used for plasma treatment (middle panel), and a picture showing plasma generation (right panel). **B**, Optical emission spectra (OES) of plasma generated using argon (Ar) gas (left graph) and air (right graph). **C**, Optical emission spectra (OES) showing the region between 280 and 340 nm where the hydroxyl (OH) radical (309 nm) peak is present.

An atmospheric-pressure non-thermal DBD plasma unit was used in this study (Fig. 1A). The plasma device consists of electrodes, dielectric and hydration prevention layers, and a magnesium oxide (MgO) layer. The electrode is 5 µm thick and 200 µm wide with a 200 µm electrode gap and a MgO layer of approximately 1 µm thick. The thickness of the silicon dioxide (SiO_2_) dielectric layer is about 30 µm and the diameter of the plasma discharge area is about 60 mm. To prevent hydration during plasma discharge, aluminum oxide (Al_2_O_3_) was added below the MgO layer. To generate plasma, air or argon (Ar) gas was injected into the device with a l liter per minute (lpm) flow rate. The average power of air and Ar plasma was 7.5 W triggered by discharge voltage and current of 0.75 kV and 80 mA, respectively.

One ml of fungal spores resuspended in saline (0.85% NaCl solution) or phosphate buffer saline (PBS) (2×10^7^ spores/ml) was placed on a petri dish (90 mm in diameter) and exposed to plasma at a distance of 5 mm (Fig. 1A). After exposure for the indicated times, spore suspensions were equally divided into 3 microcentrifuge tubes (300 µl per tube; 6×10^6^ spores) and incubated at 25°C for the indicated times. Spores exposed to gases only (air or Ar) were used as controls. Spores were also exposed to plasma treated saline or PBS. Each solution (1 ml) was treated with plasma or Ar gas (control) for the indicated times followed by the addition of 2×10^7^ spores to the treated solutions and incubated at room temperature for the indicated times.

For treatment of tomato plants, 10 tomato seedlings grown for 2 weeks were harvested from their pots and their roots were briefly washed with water. Plants were then placed in a beaker containing water and then exposed to plasma and Ar gas (control) at a distance of 1 cm from the plasma device for the indicated times. Treated plants were incubated at room temperature for 1 h and then the roots were cut and immediately frozen in liquid nitrogen. The roots were stored at −80°C until the RNA was extracted to assess the mRNA levels of target genes.

### Spore germination and structural analysis

Spores were treated with plasma or plasma treated saline and then incubated for the indicated times. Following incubation, spores were washed with saline once and resuspended in 1 ml of fresh saline, serially diluted and 100 µl spread onto PDA plates, incubated at 30°C in the dark for 2 days, and the number of spores that germinated was counted. The relative percentage of spore germination compared to that of the control (gas treated only) was calculated as follows: relative germination (%)  = (the number of germinated spores after plasma treatment/the number of germinated spores after gas treatment) x 100.

The surface and internal structures of fungal spores were analyzed using SEM and TEM, respectively, before and after plasma treatment. Spores were incubated in either saline or plasma treated saline for 10 min and then incubated for the indicated times. After incubation, fungal spores were washed with PBS twice and fixed in Karnovsky's fixative [2% (v/v) paraformaldehyde and 2% (v/v) glutaraldehyde in 1x PBS] overnight. Spores were then washed with PBS 3 times and fixed with 1% (v/v) osmium tetroxide for 2 h at room temperature in the dark. Spores were washed twice with PBS and then dehydrated by serial incubations in 30, 50, 70, 80, 90, and 100% (twice) ethanol. For SEM analysis, dehydrated spores were dried by incubating in hexamethyldisilazane (HMDS) for 15 min twice, mounted on carbon tape, coated with platinum, and then examined under a scanning electron microscope (SEM) (JEOL, Tokyo, Japan). For TEM analysis, 1 ml of 100% propylene oxide was added to the dehydrated spores and then incubated for 15 min at room temperature. After the propylene oxide was removed, a mixture (1∶1, v/v) of propylene oxide and Spurr's resin (eponate [glycerol 12 resin]: DDSA [dodecenyl succinic anhydride]: NMA [methylnadic anhydride]: DMP-30 [(2,4,6-Tris(dimethyl aminomethyl) phenol] = 29∶16∶14.3∶0.8) was added to each tube and incubated at room temperature with gentle end over end mixing for 2 h. Spores were then centrifuged at 10,000 rpm for 5 min and the resin mixture removed. Fresh Spurr's resin (1 ml) was added to the tubes that were rotated end over end overnight and the resin removed following centrifugation on the next day. Fresh Spurr's resin was added to the tubes that were then rotated end over end for 2 h. Tubes were then incubated at 70°C to induce polymerization for 8 h. The polymerized resin block was then sectioned using an ultramicrotome (Leica, Solms, Germany) and examined under TEM (JEOL, Tokyo, Japan).

For staining of lipid droplets, fungal spores were treated with Ar plasma in saline and then incubated at room temperature as described above. Spores were stained with nile red solution and examined under a fluorescence microscope as described previously [36].

### Analysis of spore death

In order to analyze apoptotic and necrotic cell death, fungal spores were stained with Anexin V and PI (Propidium Iodide) and examined by TUNEL assay after plasma treatment. Spores were treated with plasma and Ar gas (control) for 1, 5, and 10 min and then incubated at room temperature for the indicated times. After incubation, spores were washed with PBS once and incubated in 300 µl of OM buffer (1 M MgSO_4_, 8.4 mM Na_2_HPO_4_, 1.6 mM NaH_2_PO_4_) containing lysing enzyme (10 mg/ml, Sigma, St. Louis, MO, USA) at 30°C for 2 h to digest the fungal cell wall [37]. For staining with Anexin V and PI, spores were washed with PBS once and resuspened in 100 µl of 1x binding buffer. Then, 5 µl of Anexin V-FITC and PI (BD Bioscience, San Jose, CA, USA) were added to respective tubes and incubated for 15 min at room temperature in the dark followed by the addition of 400 µl 1x binding buffer. Spores were analyzed by flow cytometry (BD Bioscience, San Jose, CA, USA) and fluorescence microscopy (Nikon, Tokyo, Japan).

The TUNEL assay was conducted after the spore cell wall was digested and the spores fixed with 1 ml of 1% (v/v) paraformaldehyde (Electron Microscopy Sciences, Hatfield, PA, USA) in PBS for 15 min on ice. Spores were then processed using the APO-BrdU™ TUNEL assay Kit (Invitrogen, Molecular Probes, Eugene, NY, USA) as described by the manufacturer. After fixation, spores were washed with PBS twice and resuspended in 100 µl fresh PBS. One ml ice-cold 70% (v/v) ethanol was added and the spores incubated at −20°C for 12–18 h. Spores were washed with wash buffer twice, resuspended in 50 µl of the DNA-labeling solution (10 µl reaction buffer, 0.75 µl of dTd enzyme, 8 µl of BrdUTP, and 31.25 µl of dH2O), and then incubated at 37°C for 3 h. After incubation, spores were washed with rinse buffer twice and resuspended in 100 µl of the antibody solution (0.5 µl of the Alexa Fluor 488 dye-labeled anti-BrdU antibody with 95 µl of rinse buffer). Spores were incubated at room temperature for 30 min in the dark and then observed under a fluorescence microscope (Nikon).

### Optical emission and absorption spectroscopy

Optical emission spectroscopy (OES) was performed using a CCD spectrometer (Ocean Optics, Dunedin, FL, USA) to analyze reactive species generated following plasma treatment. Detector optical fibers (length size 600 nm) were placed near the plasma emission area and the emission spectrum monitored at wavelengths between 200–1100 nm. The spectrum data were analyzed using the SpectraSuit software (Ocean Optics).

To analyze the absorption spectra of plasma treated saline, 1 ml of saline was exposed to Ar gas (control) or plasma for 1, 5, or 10 min and then incubated at room temperature for the indicated times. After incubation, the absorption of the treated solutions was immediately monitored using a UV/visible light S-3100 spectrophotometer (Scinco, Twin Lakers, WI, USA).

### Measurement of pH, temperature, ion concentrations, and reactive species

The pH and temperature of 1 ml solutions exposed to Ar gas (control) or plasma for the indicated times were measured using a portable pH meter (Eutech Instruments, Singapore) and an infrared camera (FLIR systems Inc., Kings Hill, West Malling, UK), respectively. Ion concentrations were analyzed after filtering solutions through a 0.5 µm pore size filter, using an ion chromatograph ICS-3000 (Thermo Scientific Dionex, Sunnyvale, CA, USA).

Reactive species (hydroxyl radical, nitric oxide, and peroxynitrite) present in solution were measured using chemical indicators such as 2′,7′-dichlorodihydrofluorescein (H2DCF, major species; hydroxyl radical and peroxynitrite), 4-amino-5-methylamino-2′,7′-difluorofluorescein (DAF-FM, major species; nitric oxide), and terephthalic acid (TA, major species; hydroxyl radical). H2DCF was obtained by the hydrolysis of 2′,7′-dichlorodihydrofluorescein diactate (H2DCFDA) in which H2DCFDA was incubated in 0.05 M NaOH for 30 min at 37°C in the dark [38]. One ml of saline containing H2DCF and DAF-FM (10 µM, Life technologies, Washington DC, USA) was treated with Ar gas (control) or plasma for the indicated times. Solutions were then transferred to a black walled 96-well assay plate (Corning, NY, USA) (100 µl in each well), and the plate was read at 495/515 (ex./em.) nm using a microplate reader (Biotek, Winooski, VT, USA). Hydroxyl radical was also measured using terephthalic acid (Sigma). Saline (1 ml) containing terephthalic acid (20 mM) was exposed to Ar gas (control) or plasma for the indicated times and the fluorescence of treated solutions was measured at 310/425 (ex./em.) nm [39].

To measure the level of intracellular reactive species, fungal spores in saline (2×10^7^/ml) were treated with Ar gas or plasma at the indicated times and then incubated for the indicated times at room temperature. After incubation, spores were washed with PBS and resuspended in fresh PBS containing chemical indicators (final concentration 10 µM), H2DCFDA (major species; hydroxyl radical and peroxynitrite), DAF-FMDA, and APF (2-[6-(4′-Amino) phenoxy-3H-xanthen-3-on-9-yl] benzoic acid, major species; hydroxyl radical and hypochlorite (OCl^−^)). After incubation at room temperature for 1 h in the dark spores were washed with PBS twice and resuspened in100 µl of fresh PBS. Spore suspensions were then transferred to a black walled 96-well assay plate and the plate was read at 495/515 (ex./em.) nm using a microplate reader.

### Quantitative PCR analysis

To measure the mRNA levels of apoptosis related genes expressed by fungal spores, spores were treated with Ar gas (control) or plasma for 10 min and then incubated for 0 and 2 h. After incubation, spores were washed with PBS twice, dried in a vacuum container, and then stored at −80°C until total RNA was extracted using the TaKaRa RNAiso Plus kit (TaKaRa Bio, Tokyo, Japan) as described by the manufacturer. The RNA concentration was measured using a nanodrop (Biotek) and then 1 µg RNA was treated with RNase-free DNase (Promega, Madison, WI, USA) at 37°C for 1 h to remove genomic DNA. The same amount of RNA (120 ng) was used to synthesize cDNA using the miScriptII PCR System following the manufacturer's protocol (Qiagen, Valencia, CA, USA). PCR conditions were as follows: 60 min at 37°C and then 5 min at 95°C. Apoptosis related genes including dinamin (Dnm), metacaspase, and apoptosis inducing factor (AIF) were amplified and quantified at every thermal cycle using iQ SYBR Green Supermix (BioRad) and the CFX96™ real time RT-PCR system (Bio-Rad). The relative mRNA expression levels were expressed as a ratio of the expression level of a reference gene (18S ribosomal RNA) as previously described [40]. The cycle threshold (Ct) was determined and the difference in the Ct value between the plasma exposed sample and the control (Ar gas treated) was used to calculate the relative target gene expression level as follows: Ratio  = (2)^ΔCt target (control-sample)^/(2)^ΔCt reference (control-sample).^

To determine the induction of plant resistance genes following plasma exposure, the mRNA expression levels of PR genes in the roots of the susceptible tomato (*S. lycopersicum*) cultivar (Titichal) were measured by real-time RT-PCR. Tomato plant leaves were exposed to plasma and then roots were collected and frozen in liquid nitrogen as described above. Roots were macerated into powder in liquid nitrogen and total RNA was isolated using the TaKaRa RNAiso Plus kit (TaKaRa Bio). Samples were treated with DNase and real time RT-PCR was carried out as described above. Sequences of all primers used are listed in Table 1.

**Table 1:** List of primers used for quantitative PCR analyses.

No.	Sequence	Primer Details
**Apoptosis related genes in ** ***F. oxysporum***		
1	GGCAACATTGTCATGTCTGG	Actin_Forward
2	TTGGAGATCCACATCTGCTG	Actin_Reverse
3	CTTGGTTTTATCGGCGTTGT	Dynamin GTPase_Forward
4	AAACGCTCACGGATATGACC	Dynamin GTPase_Reverse
5	TGCATCTACCCTGTTGACCA	Metacaspase 1_Forward
6	CAGCTTCCTTGGCTAGGTTG	Metacaspase 1_Reverse
7	CTCACCGATGATCAGCAAAA	Metacaspase 2_Forward
8	CGGGTAAATGACTTCGTCGT	Metacaspase 2_Reverse
9	CCAAATACGGCGACAAGTTT	Apoptosis inducing factor 1_Forward
10	ACATAGTCCCTGGTCGCATC	Apoptosis inducing factor 1_Reverse
11	GATGCGACCAGGGACTATGT	Apoptosis inducing factor 2_Forward
12	GGCCAGCATTAATCAGGGTA	Apoptosis inducing factor 2_Reverse
**Pathogenesis related (PR) genes of ** ***S. lycopersicum***		
1	TGACGGAGAATTAGGGTTCG	18S rRNA_Forward
2	CCTCCAATGGATCCTCGTTA	18S rRNA_Reverse
3	TGGGTTGATGAGAAGCAATG	PR1a_Forward
4	AAAGTACCACCCGTTGTTGC	PR1a_Reverse
5	CCAAGACTATCTTGCGGTTC	PR1b_Forward
6	GAACCTAAGCCACGATACCA	PR1b_Reverse
7	TATAGCCGTTGGAAACGAAG	Acidic glucanase PR2a_Forward
8	TGATACTTTGGCCTCTGGTC	Acidic glucanase PR2a_Reverse
9	CAACTTGCCATCACATTCTG	Basic glucanase PR2b_Forward
10	CCAAAATGCTTCTCAAGCTC	Basic glucanase PR2b_Reverse
11	CAATTCGTTTCCAGGTTTTG	Chitinase 3 acidic PR3a_Forward
12	ACTTTCCGCTGCAGTATTTG	Chitinase 3 acidic PR3a_Reverse
13	AATTGTCAGAGCCAGTGTCC	Chitinase 9 basic PR3b_Forward
14	TCCAAAAGACCTCTGATTGC	Chitinase 9 basic PR3b_Reverse
15	AATTGCAATTTTAATGGTGC	Osmotin-like PR5_Forward
16	TAGCAGACCGTTTAAGATGC	Osmotin-like PR5_Reverse

### Statistical analysis

All experimental values are expressed as the mean ± standard deviation of replicates (≥3). Statistical analysis of the data was performed using the Student's *t* test to determine significance between data points and significant differences were established at *p*<0.05 or *p*<0.01 (*denotes p<0.05 and ** denotes p<0.01).

## Results

### Spore germination decreases over time following Ar plasma treatment

Plasma generated from argon (Ar) and air (Fig. 1A) produced various reactive species identified following optical emission spectroscopy (OES) analysis (Fig. 1B). The optical emission spectra analysis identified several reactive oxygen species of biological importance including the hydroxyl (OH) radical (309 nm) and singlet oxygen (778 nm) in Ar plasma (Fig. 1B and C). However, peaks corresponding to the hydroxyl radical or singlet oxygen were not observed in air plasma (Fig. 1B and C). Peaks between 690–900 nm (Ar plasma) and 300–400 nm (air plasma) correspond to light emitted when Ar (in Ar plasma) and N_2_ (in air plasma) molecules are excited by high energy (electric voltage) and then return to their ground states (Fig. 1B). Excited Ar and N_2_ species are involved in generating ROS and RNS (hydroxyl radical and singlet oxygen in our case) by exciting molecules in ambient air and may therefore not have an impact on fungal spore viability (Fig. 1B).

**Figure 2:**
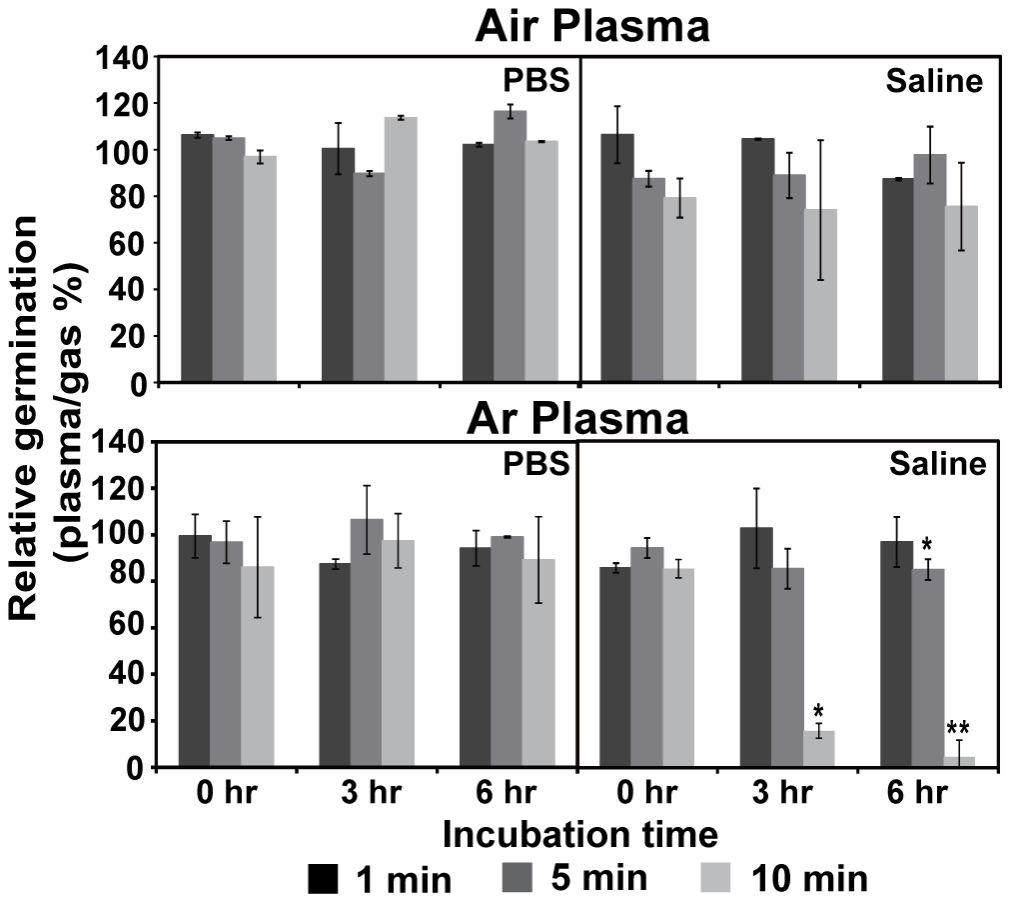
Germination of fungal spores after plasma treatment. Relative germination of *F. oxysporum f.sp.lycopersici* spores was assessed after air and argon plasma treatment in PBS or saline. The relative spore germination percent was calculated as follows: (number of germinated spores treated with plasma/number of germinated spores treated with gas only) x 100. *p<0.05 and **p<0.01; Student's *t* test.

After fungal spores were exposed to Ar or air plasma in PBS or saline (0.85% NaCl solution), changes in spore germination were not immediately apparent (0 h) (Fig. 2). However, when spores were incubated in solution after plasma exposure, spore germination in saline after Ar plasma treatment for 10 min (but not for 1 or 5 min) was decreased over time (Fig. 2). Germination of spores treated in PBS or with air plasma was not significantly affected (Fig. 2). The temperature of both saline and PBS was maintained between 20–28°C during plasma treatment and should not affect spore germination (Fig. S1). These results indicate that fungal spores incubated in saline were inactivated over time following exposure to Ar plasma.

**Figure 3:**
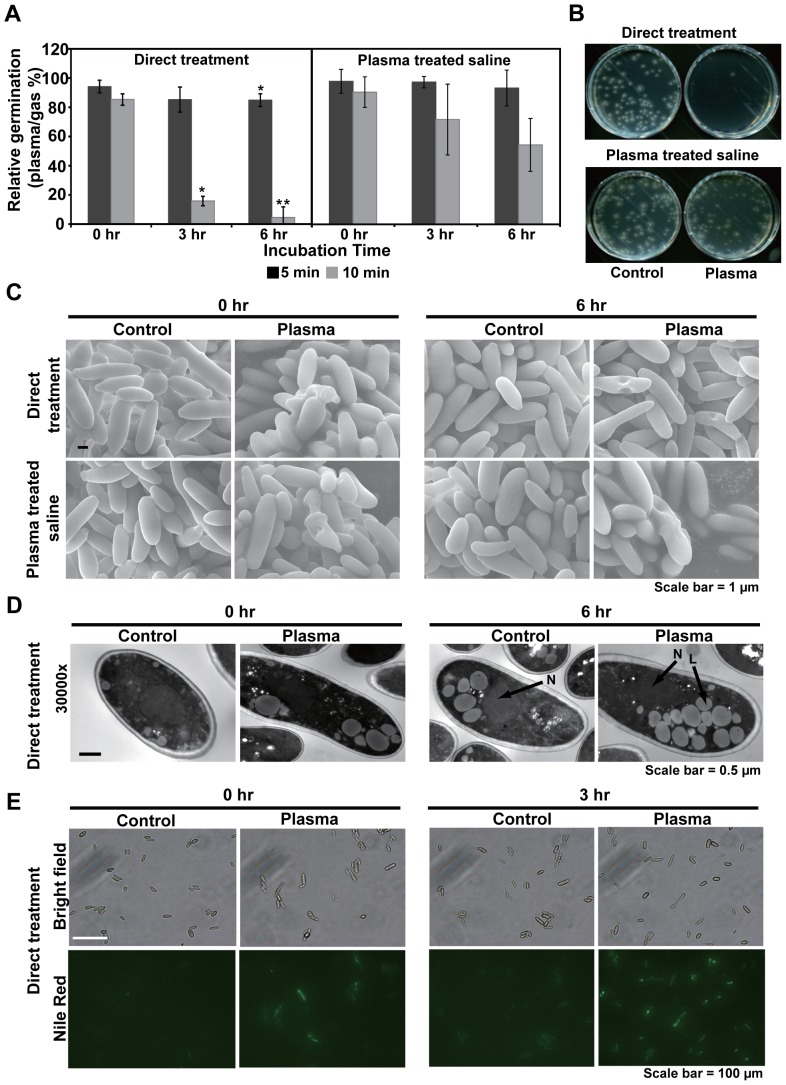
Effect of direct plasma and plasma-treated saline on spore germination and structure. **A**, Relative spore germination rates observed during the incubation period following treatment with direct Ar plasma (left graph) or Ar plasma treated saline (right graph). The relative spore germination percent was calculated as described in Figure 1. *p<0.05 and **p<0.01; Student's *t* test. **B**, Fungal spores grown on PDA plates after treatment with direct Ar plasma or Ar plasma treated saline. **C**, Surface morphology of fungal spores analyzed by SEM after plasma treatment. **D**, Ultrastructure of fungal spores analyzed by TEM after plasma treatment. Lipid droplets (L) are indicated. **E**, Spores stained with nile red solution after plasma treatment.

Reduction in germination rates was also observed when spores were incubated in Ar plasma treated saline (Fig. 3A and B). Spores added to saline first treated with Ar plasma for at least 10 min (but not 5 min) decreased germination rates over incubation time (Fig. 3A). Germination of spores treated in saline with Ar plasma for 10 min was more impaired compared to that of spores incubated in plasma-treated saline for the same exposure time (Fig. 3A and B) indicating that factors resulting from direct plasma treatment may generate additional toxic effects.

Analysis of spore morphology by SEM did not identify significant structural changes following exposure to either direct Ar plasma or exposure to Ar plasma-treated saline although a few crushed spores were observed (Fig. 3C). When spores were examined by TEM, changes to internal structures were not observed following direct plasma treatment (Fig. 3D). However, an increased number of lipid droplet like bodies in the cytoplasm of spores treated with plasma were observed after a 6 h incubation (Fig. 3D). In order to examine if lipid droplets were accumulated after plasma treatment, spores were stained with nile red (staining lipid droplets). The number of spores emitting nile red fluorescence increased after a 3 h plasma treatment (Fig. 3E) indicating that the structure observed in TEM analysis might be lipid droplets and they were accumulated upon plasma exposure.

### Ar plasma induced both necrosis and apoptosis in fungal spores

**Figure 4:**
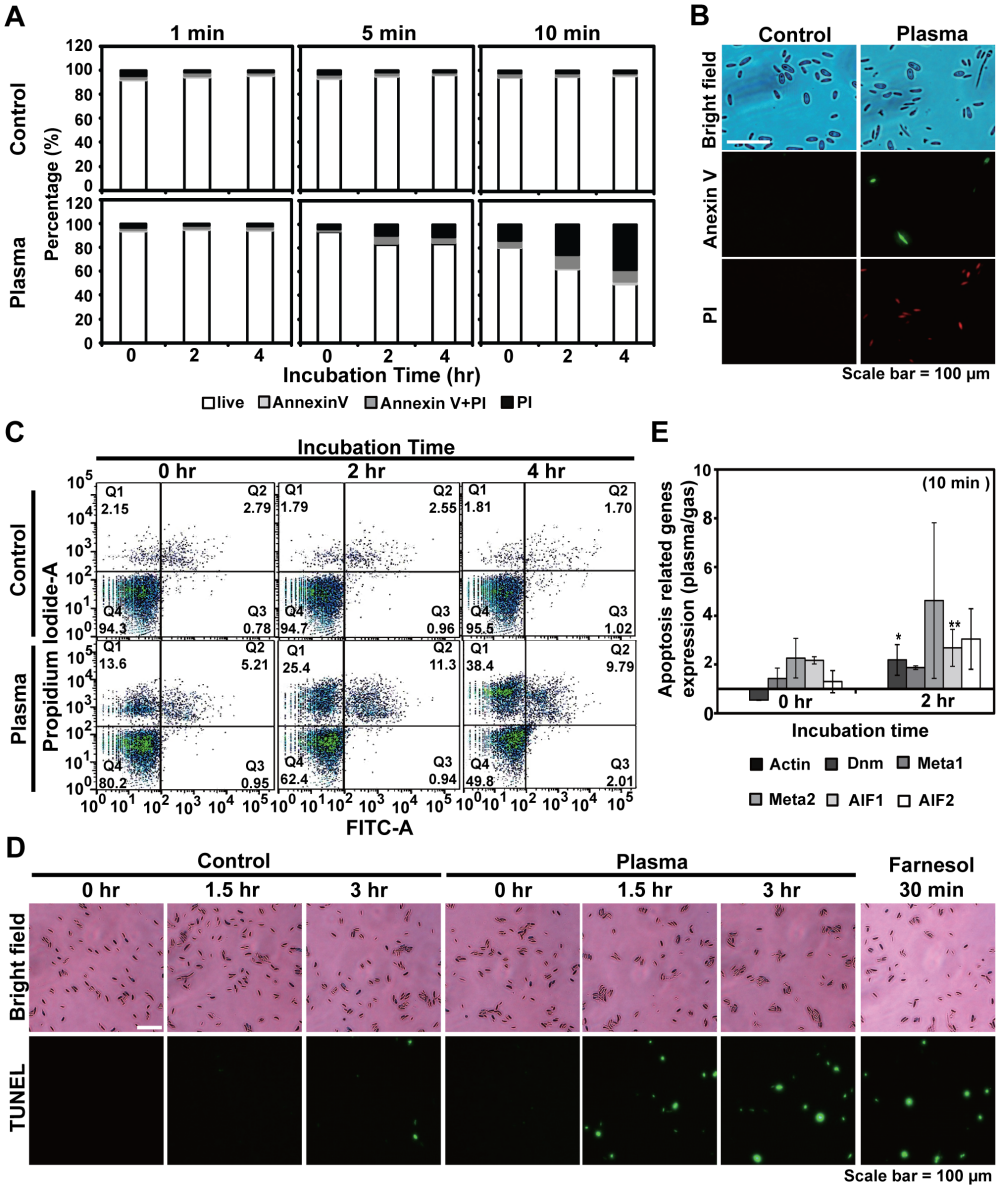
Mechanisms associated with spore death. **A**, Relative comparisons of apoptotic and necrotic death indicated by the percentage of Annexin V and PI stained spores following Ar plasma treatment over time. **B**, Image of spores stained with Annexin V and PI after a 10 min plasma treatment. **C**, Proportion of spores stained with Annexin V and PI analyzed by flow cytometry. Spores were treated with Ar plasma in saline for 10 min and then incubated for 0, 2, and 4 h. **D**, mRNA expression levels of the apoptosis related genes Dnm, Meta1, 2, and AIF1, 2 were analyzed by real time RT-PCR. Spores were treated with Ar plasma in saline for 10 min and then incubated for 0 and 2 h. Each value represents the average of 3 replicate measurements. *p<0.05 and **p<0.01; Student's *t* test. **E**, Spores treated with plasma for 10 min were subjected to TUNEL assay. Fungal spores treated with farnesol (0.5 mM) to induce apoptosis [47] were used as a positive control.

Reduced germination rates were indicative of either spore death or stasis. The observed increase in lipid droplets in the cytoplasm of spores exposed to plasma is suggestive of apoptosis since lipid droplets are known to accumulate during apoptosis [41]. To determine the mechanism of inactivation induced following exposure to Ar plasma, spores were stained with Annexin V and PI (propidium iodide). Exposure of spores to Ar plasma for 1 min did not result in Annexin V or PI staining suggesting that cell death at this point was not taking place (Fig. 4A). After 5 and 10 min, however, more spores exhibited PI fluorescence indicative of necrotic death and the proportion of necrotic spores increased with time (Fig. 4A). A few spores treated with plasma for 5 or 10 min stained Annexin V positive and the number of Annexin V positive cells increased over time (Fig. 4A and B). Although plasma treatment induced apoptosis in fungal spores, the majority of cell death was due to necrosis (Fig. 4A). These results demonstrate that the observed reduction in spore germination over time may be a consequence of spore death (necrotic and apoptotic death) resulting from plasma treatment.

Interestingly, we observed apoptosis (indicated by Annexin V staining) in spores treated with plasma for 5 and 10 min (Fig. 4 A and B). Apoptosis rates were increasing over time with the majority of these spores in the later stages of apoptosis (Fig. 4C). Over a 2 h incubation following a 10 min plasma treatment, the number of spores in the later stages of apoptosis increased (Fig. 4C). Induction of apoptosis was further examined by assessing the expression levels of genes associated with apoptosis such as dynamin GTPase (Dnm), metacaspase, and apoptosis inducing factor (AIF). The Dnm and AIF1 mRNA levels were significantly elevated over a 2 h period following a 10 min plasma treatment (Fig. 4D). TUNEL assay results showed that the number of spores with breaks in DNA (indicative of apoptosis) increased during incubation after 10 min plasma treatment (Fig. 4E).

### Increases in RNS and nitrite in saline may be responsible for spore death

**Figure 5:**
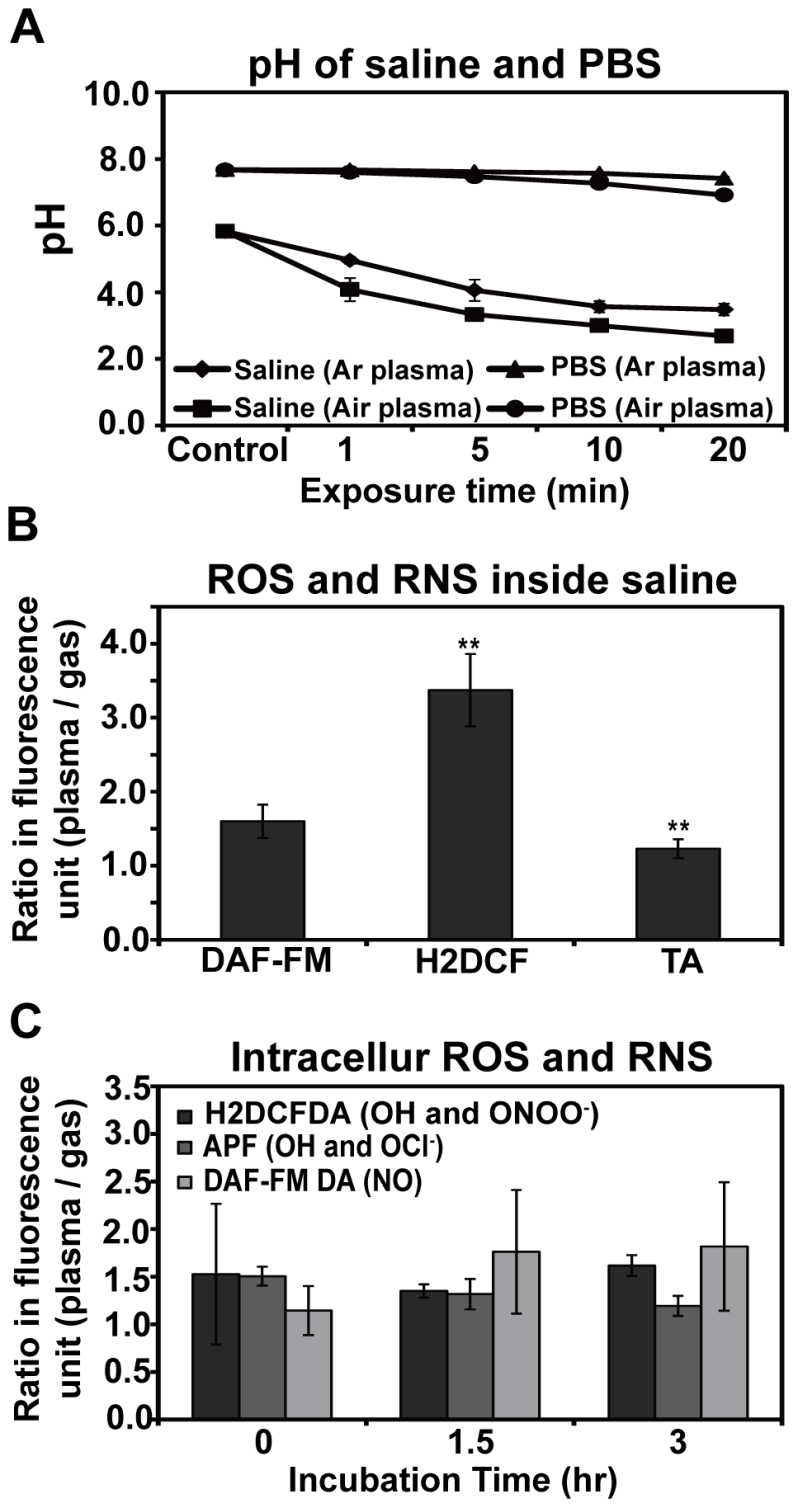
pH measurements and reactive species levels. **A**, pH of PBS and saline after treatment with air or argon plasma. Each data point represents the average of 3 replicate measurements. **B**, Levels of ROS and RNS in saline after plasma treatment for 10 min. **C**, ROS and RNS levels inside fungal spores after plasma treatment for 10 min and during consecutive incubations of 0, 1.5, and 3 h.

To identify factors responsible for the induction of apoptotic and necrotic death, the pH of the solution following plasma exposure was first determined. Following a 10 min exposure to Ar plasma decreased the pH to 3–4 (Fig. 5A). Saline treated with air plasma exhibited a slightly greater decrease in pH over time compared to changes in pH induced following Ar plasma treatment (Fig. 5A). No apparent changes in pH were observed to PBS following treatment with either Ar or air plasma (Fig. 5A). The decrease in pH observed following treatment with either Ar or air plasma may have played a role in killing fungal spores, however, no significant spore inactivation was observed in saline treated with air plasma (Fig. 2 and Fig. 5A) suggesting that decreasing the pH was likely not responsible for the observed death.

The level of hydroxyl (OH) radicals in saline measured using TA (terephthalic acid) was slightly increased after plasma treatment (Fig. 5B) and saline ROS levels (measured using H2DCF) were increased 3-fold following plasma exposure (Fig. 5B). Since major ROS species detected by H2DCF are hydroxyl radical and peroxynitrite (ONOO^−^) and the level of hydroxyl radical measured by TA was slightly increased, the ROS levels detected by H2DCF were mostly likely due to the presence of peroxynitrite. Nitric oxide (NO) levels (detected by DAF-FM) were more than 1.5-fold greater following plasma treatment compared to the control (Ar gas treatment) but the difference was not significant (Fig. 5B), suggesting that peroxynitrite production in saline following Ar plasma exposure may be a factor associated with spore death.

Intracellular ROS and RNS levels were also measured following plasma treatment. After a 10 min plasma treatment the relative intracellular nitric oxide levels (measured by DAF-FMDA) continuously increased over time (up to1.8-fold higher after 3 h) (Fig. 5C) but not significantly (Fig. 5C). Compared to nitric oxide levels, significant change in the levels of other species (such as the hydroxyl radical, peroxynitrite, or hypochlorite) was not observed (Fig. 5C).

**Figure 6:**
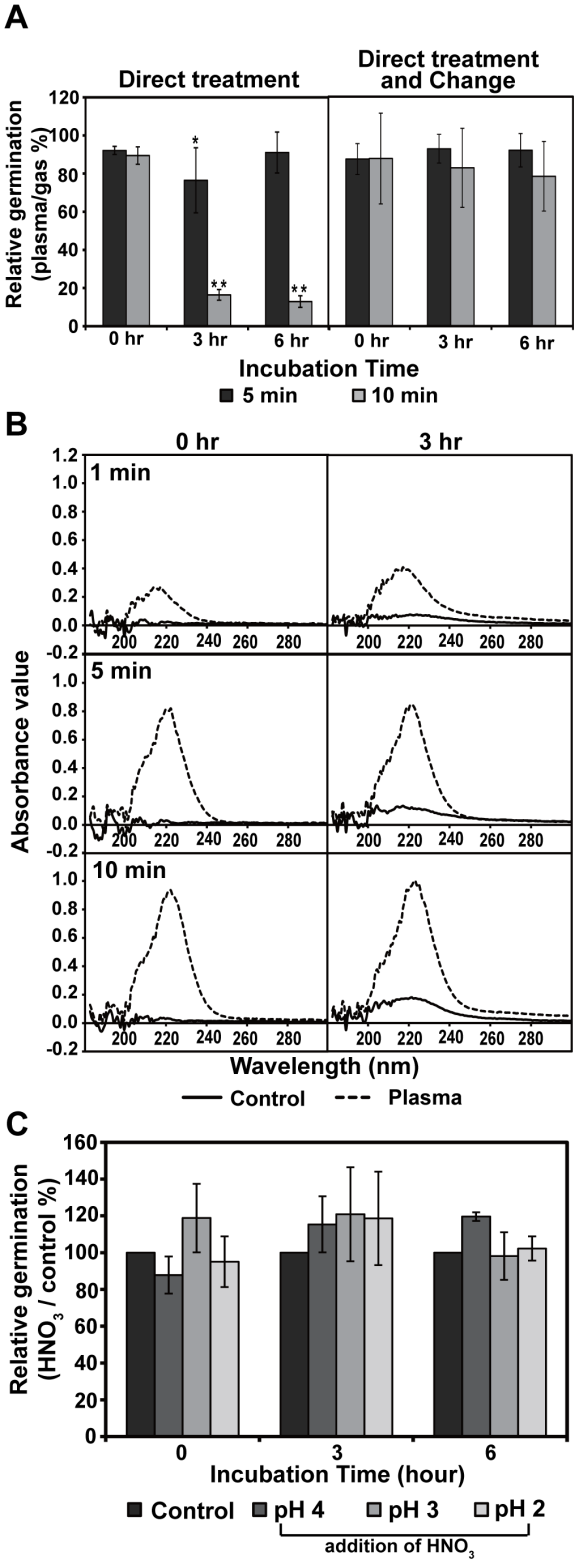
Effect of solution change and absorption spectroscopy analysis. **A**, Relative spore germination after Ar plasma treatment and consecutive incubation without (left graph) or with (right panel) a solution change. The calculations for relative germination and statistical analysis were performed as described in Figure 2. **B**, Absorption spectra of saline treated with Ar gas (control) or plasma for 1, 5, or 10 min and then incubated for 0 and 3 h. **C**, Relative spore germination in saline solution with the pH adjusted using nitric acid (HNO_3_).

Additional chemical changes taking place in spores following Ar plasma treatment in saline were determined by transferring treated spores to fresh saline solution following plasma treatment. No significant changes in spore germination were observed when spores were transferred to fresh saline compared to the reduced germination rate observed for spores that remained in Ar plasma-treated saline over time (Fig. 6A). This indicated that changes to the saline solution resulting from Ar plasma treatment were responsible for the observed spore death. Analysis of the absorption spectra of Ar plasma-treated saline identified peak(s) around 220 nm and the height of the peak(s) increased following longer plasma treatments and during the subsequent incubation period (Fig. 6B). The 220 nm peak was suggestive of nitrate (NO_3_^−^) and nitrite (NO_2_^−^) based on previous reports [42], [43]. Our ion analysis data also showed that NO_2_^−^ and NO_3_^−^ ions were generated in Ar plasma treated saline with increased levels observed following extended treatment times (Table 2). It is possible that nitrate and nitrite are responsible for the increased rates of fungal spore death observed during the incubation period. However, spores incubated in saline supplemented with HNO_3_ (nitric acid) did not show any significant differences in germination during the incubation period compared to the control (saline only) (Fig. 6C) indicating that nitrite may have played a more significant role in fungal spore inactivation.

**Table 2:** Ion analysis of saline exposed to plasma.

Ions		Ar treated (mg/L)				Plasma treated (mg/L)			
		1 min	5 min	10 min	20 min	1 min	5 min	10 min	20 min
Cation	Na	3201.89	3200.73	3286.13	3314.47	3167.19	3205.65	3277.49	3366.01
	NH_4_	13.40	14.03	50.30	13.23	11.73	12.38	12.50	12.26
	K	4.05	5.45	9.48	4.64	3.62	4.54	4.50	4.41
	Mg	2.58	2.10	2.25	1.73	1.93	0.00	1.87	1.84
	Ca	14.20	11.53	10.68	9.87	10.04	9.59	10.30	12.12
Anion	Cl	5339.74	5537.16	5507.83	5620.24	5357.07	5416.04	5548.76	5678.52
	SO_4_	3.53	3.74	3.65	3.77	3.55	3.54	3.67	3.97
	NO_2_	0.00	0.00	0.00	0.00	5.63	13.02	19.02	21.29
	NO_3_	3.38	3.70	3.58	3.97	3.92	10.25	20.68	43.95

### Host plant resistance can be induced by plasma treatment

**Figure 7:**
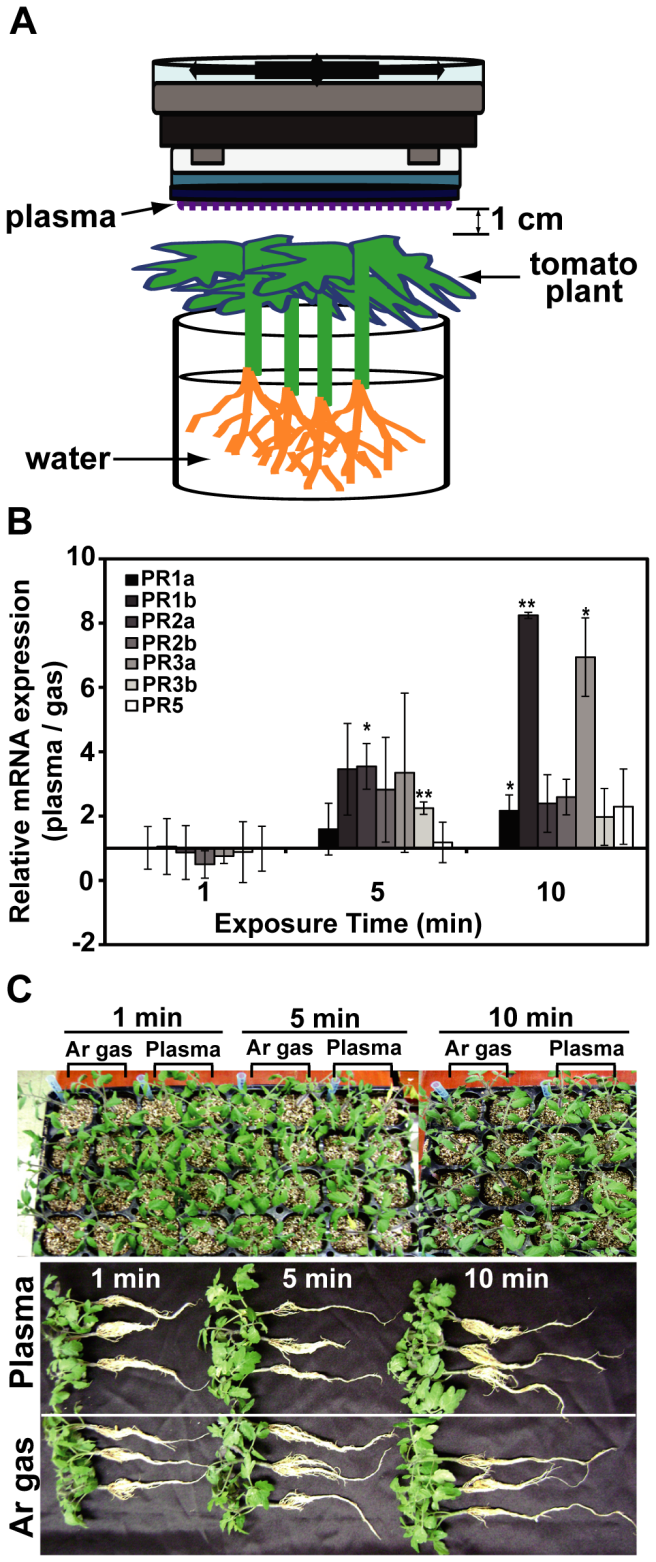
Expression of resistance genes in tomato plants after plasma treatment. **A**, Experimental setup for treating tomato plants with Ar (argon) plasma. The distance between plant leaves and the plasma emission area was approximately 1 cm. **B**, The relative mRNA levels for 7 PR genes expressed in roots after plasma treatment. Each value represents the average of 3 replicate measurements, *p<0.05 and **p<0.01; Student's *t* test. **C**, Tomato plants grown for 2 weeks after treatment with Ar gas (control) or plasma.

Plants respond to pathogens by up-regulating expression of resistant genes [44]. To test the potential of plasma to induce the up-regulation of disease-resistance genes we examined changes to the expression levels of pathogenesis related (PR) genes in a tomato plant cultivar susceptible to *F. oxysporum* after plasma treatment. PR genes are known to be expressed in response to infection [44]–[46]. One hour after tomato plants were exposed to plasma (Fig. 7A), the mRNA levels of 7 PR genes (Table 1) were measured in roots (the target organ for *F. oxysporum*). Expression of all PR genes was detected after a 5 min treatment (but not after a 1 min exposure) (Fig. 7B). Plasma treatment for 10 min significantly up-regulated PR1a, PR1b, and PR3a (chitinase) mRNA expression levels compared to levels observed following treatment for 1 and 5 min (Fig. 7B). Plants treated with plasma for 10 min grew in similar fashion as Ar gas treated plants (control) suggesting that plasma treatment did not seem to cause any damage to tomato plants (Fig. 7C). These results suggested that exposing plants to plasma up-regulated resistant gene expression in the roots without causing any damage to the plants.

## Discussion

The present study demonstrated that exposure to Ar plasma in saline (0.85% NaCl solution) induced the continuous inactivation of *F. oxysporum* spores. The microbicidal effects of plasma treated NaCl solution was previously demonstrated and it was due to chemical alterations to the NaCl solution after plasma treatment [47]. In our study, significant changes in spore viability were not apparent immediately following plasma treatment, however, spore inactivation was apparent over time. Specifically, exposure to plasma for 10 min was associated with the most effective degree of continuous spore inactivation.

The search for factor(s) associated with continuous spore inactivation identified changes in the saline composition following exposure to Ar plasma rather than the observed saline acidification since lowering the pH in the absence of Ar plasma treatment did not significantly change the percentage of spores germination. The elevated levels of peroxynitrite and the hydroxyl radical in saline following plasma treatment likely played a role in spore inactivation. In addition, nitrite was also a toxic factor. Nitrite might be responsible for reducing the pH of the saline solution but we had already confirmed that acidification (pH 3) following the addition of nitric acid (HNO_3_) did not negatively affect spore germination (Fig. 6C). Antimicrobial effect of nitrite was previously reported [48]. As demonstrated previously, chemical changes to saline after plasma treatment may be responsible for the death of fungal spores during the incubation following Ar plasma exposure [47].

Interestingly, we observed an increase in the number of fungal spores at the later stages of apoptosis during the incubation period following plasma treatment (in saline). Studies have shown that various factors including ROS and chemicals like farnesol and tomatine can induce fungal apoptosis-like death [32], [37], [49], [50]. Since plasma can generate reactive species and modulate the level of intracellular and extracellular reactive species, apoptosis-like cell death of fungi may have been induced by plasma treatment. Although the proportion of apoptotic fungal spores was low compared to that of spores undergoing necrotic death, our data suggested that plasma exposure could also control fungal diseases by activating pathways resulting in apoptosis.

A remarkable outcome from the present study was the observation that control of fungal diseases by plasma treatment could also be mediated by up-regulating host resistance genes. This multi-faceted effect of plasma may be due to specific dynamics associated with reactive species (resulting from plasma treatment). Although additional research is needed to define the respective effects of the different reactive species from plasma, two implications can be considered for effective disease control. First, same dose of plasma can produce contradictory effects on fungal pathogen (inactivation) and host plant (activation of resistance). This potential of plasma can be usefully applied to developing the indoor culture system. However, we observed the plasma effect on fungal spore and host plant separately, not in mixed culture of them. Pathogen invasion is known to trigger increased production of reactive species within host cell [51]–[53]. Since the microenvironment in plant cell is much more complex than sodium chloride solution used in our experiments, plasma may possibly modulate the level of reactive species and chemical environment within plant cell infected with fungal pathogen through interaction with microenvironment. This may result in different antifungal activity of plasma in plant compared to that observed in *in vitro* culture. Further investigation may be needed for understanding plasma effect on in planta fungal infection. Second, plasma can act like pathogen attack in activating host defense systems. The mRNA levels for several PR genes normally activated in response to pathogen infection were significantly increased in tomato cultivars susceptible to *F. oxysporum* following plasma treatment. Plasma treatment did not affect plant health (or growth) and gene up-regulation may have been due to the production of ROS and RNS that have been shown to activate plant resistance mechanisms [51]–[53]. Of great interest was that plant leaves were exposed to plasma but induction of resistance genes was observed in roots, suggesting that a signal triggered by plasma was transferred from the leaves to the roots resulting in the activation of resistance gene expression. The specific mechanisms behind these processes remain to be elucidated.

## Conclusions

Data presented in this report demonstrated that the same dose of plasma had the potential of inactivating fungal spores and activating host PR genes without affecting plant growth. In addition, plasma triggered fungal spore death over time. These data suggested that plasma represents a novel technology with the capacity of inactivating pathogens while simultaneously up-regulating resistance genes in plants.

## Supporting Information

Figure S1 **Temperature of solutions after Ar and air plasma treatment.** Temperature of saline and PBS after Ar and air plasma treatment.
(TIF)
