# Supplementary figures and images for: Non-Thermal Plasma Treatment Diminishes Fungal Viability and Up-Regulates Resistance Genes in a Plant Host
Source: PLoS One. 2014 Jun 9;9(6):e99300. doi: 10.1371/journal.pone.0099300 (PMC4049833; doi:10.1371/journal.pone.0099300)

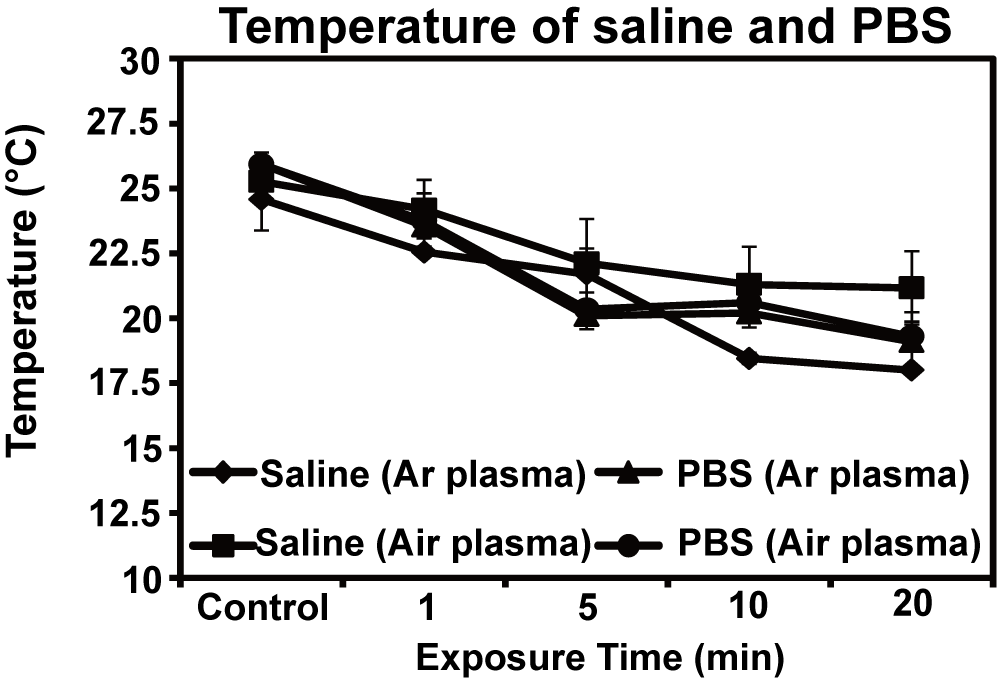

Supplement: Figure S1 — Temperature of solutions after Ar and air plasma treatment. Temperature of saline and PBS after Ar and air plasma treatment. (TIF) [file pone.0099300.s001.tif]
